# Effects of Fast Simple Numerical Calculation Training on Neural Systems

**DOI:** 10.1155/2016/5940634

**Published:** 2016-01-06

**Authors:** Hikaru Takeuchi, Tomomi Nagase, Yasuyuki Taki, Yuko Sassa, Hiroshi Hashizume, Rui Nouchi, Ryuta Kawashima

**Affiliations:** ^1^Smart Ageing International Research Center, Institute of Development, Aging and Cancer, Tohoku University, Sendai 980-8575, Japan; ^2^Faculty of Medicine, Tohoku University, Sendai 980-8575, Japan; ^3^Division of Developmental Cognitive Neuroscience, Institute of Development, Aging and Cancer, Tohoku University, Sendai 980-8575, Japan; ^4^Department of Functional Brain Imaging, Institute of Development, Aging and Cancer, Tohoku University, Sendai 980-8575, Japan

## Abstract

Cognitive training, including fast simple numerical calculation (FSNC), has been shown to improve performance on untrained processing speed and executive function tasks in the elderly. However, the effects of FSNC training on cognitive functions in the young and on neural mechanisms remain unknown. We investigated the effects of 1-week intensive FSNC training on cognitive function, regional gray matter volume (rGMV), and regional cerebral blood flow at rest (resting rCBF) in healthy young adults. FSNC training was associated with improvements in performance on simple processing speed, speeded executive functioning, and simple and complex arithmetic tasks. FSNC training was associated with a reduction in rGMV and an increase in resting rCBF in the frontopolar areas and a weak but widespread increase in resting rCBF in an anatomical cluster in the posterior region. These results provide direct evidence that FSNC training alone can improve performance on processing speed and executive function tasks as well as plasticity of brain structures and perfusion. Our results also indicate that changes in neural systems in the frontopolar areas may underlie these cognitive improvements.

## 1. Introduction

This study focused on training to improve performance on a fast simple numerical calculation (FSNC) task, which involves quickly solving mathematical problems, namely, single-digit addition, subtraction, and multiplication. The ability to complete an FSNC task correlates with processing speed, quantitative ability or knowledge, and general intelligence [1]. Previous studies of psychological interventions showed that cognitive interventions involving arithmetic [2, 3] or FSNC [4] tasks lead to improvements in performance on untrained cognitive tasks (transfer effects) among the elderly as well as dementia patients.

However, some questions related to these studies remain to be answered. First, these studies used multiple training protocols such as reading and arithmetic involving simple and more complex numerical calculations [2, 3] or a battery of several cognitive training tasks including FSNC [4]. Thus, whether FSNC training alone affects untrained cognitive functions remains unclear. Second, whether the same effect occurs in the young remains to be investigated. Third, the effects on neural systems are also unclear.

This study aimed at investigating the effect of FSNC training on cognitive functions and neural systems in healthy young adults. Considering the transfer effects brought about by the FSNC training, it is important to investigate the extent of and neural mechanisms underlying these FSNC training effects. Among FSNC training-affected neural mechanisms, we focused on changes in regional gray matter volume (rGMV) and regional cerebral blood flow during rest (resting rCBF). Using imaging analyses, we determined whether the effects of FSNC training extend beyond task-specific functional activation in the brain and, if so, the areas in which such changes occur.

Based on the results of our previous study [4], we hypothesized that FSNC training would improve executive functions and processing speed and activate neural mechanisms in the prefrontal cortex. In that study, we found that participation in a brain training game that included FSNC improved subsequent performance on processing speed and speeded executive function tasks. Several prefrontal regions, including areas in the middle frontal, inferior frontal, orbitofrontal, and frontopolar areas, are activated during numerical calculation [5, 6] and executive functioning and are also associated with processing speed (e.g., [7]). Thus, consistent with the hypothesis of our previous study [4], we reasoned that neural mechanisms in the prefrontal regions would be affected by FSNC training.

**Figure 1:**
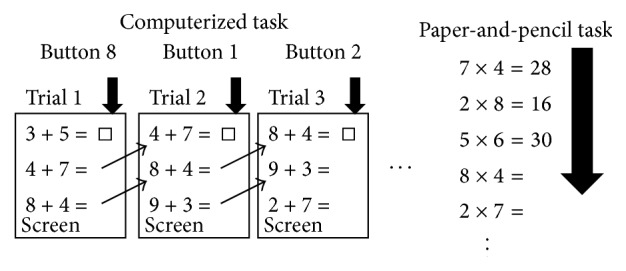
Schema of examples of training tasks used in this study. Training tasks consisted of computerized (addition, subtraction, and multiplication) and paper-and-pencil (addition, subtraction, and multiplication) tasks.

Using (a) various psychological measures, such as arithmetic measures, processing speed, and executive function, (b) rGMV analysis with voxel-based morphometry (VBM), and (c) resting rCBF analyses, we investigated the effects of 1-week intensive (up to 4 h/day) adaptive FSNC training on these variables in healthy young adults. Training consisted of three paper-and-pencil FSNC tasks (addition, subtraction, and multiplication) as well as three computerized FSNC tasks (Figure 1). We included three different operations (addition, subtraction, and multiplication) and two formats (a computerized format and a paper-and-pencil format) to increase transfer effects. This reasoning is because, as a general rule, heterogeneous training programs are thought to strengthen transfer effects [8, 9].

## 2. Methods

### 2.1. Participants

The study reported here was implemented in conjunction with and shared control group participants with our previous study which investigated the effects of training on processing speed [10]. Sixty-three healthy, right-handed university or postgraduate students (32 men, 31 women) participated in this study. Their mean age was 21.6 years [standard deviation (SD), 1.68]. Of these 63 subjects, 23 were assigned to a processing speed training group for another study [10], and the data from the remaining 40 subjects were used in the FSNC group or the nonintervention control group. All participants had normal vision, and none had a history of neurological or psychiatric illness. The latter was assessed with our laboratory's routine questionnaire about whether they had or have certain illnesses. Handedness was evaluated using the Edinburgh Handedness Inventory [11]. Each subject provided written informed consent according to the Declaration of Helsinki (1991). The Ethics Committee of Tohoku University approved the study.

Group assignments were performed in the following manner as described below. In 3 months, there were six experimental periods, each lasting 8 days. Among these six periods, the first, third, and fifth experimental periods involved PS training and no intervention. The rest of the experimental periods involved FSNC training and no intervention. The periods for the FSNC (and the nonintervention control group) and the periods for training on processing speed (and the nonintervention control group) were rotated. We could perform one type of intervention in one experimental period because of the limitation of several types of experimental resources. Subjects chose which period they participated at, but they did not know there were two types of training in the experiment. They were randomly assigned to intervention groups or the no-intervention group. Therefore, neither subjects nor experimenters could decide which groups subjects could be assigned to, and distribution amongst the three groups was arbitrary.

The FSNC training group comprised 19 participants (9 men, 10 women; mean age, 21.4 years; SD 1.8). The no-intervention group comprised 21 participants (12 men, 9 women; mean age, 21.2 years; SD 1.7). Participants in the training and no-intervention groups did not differ significantly (*P* > 0.1, two-tailed *t*-tests) in basic background characteristics such as age, sex, and scores on Raven's Advanced Progressive Matrix [12], which measures cognitive ability central to general intelligence [13], and scores for simple arithmetic tasks. One subject in both the training and no-intervention groups was not able to participate in post-MRI and psychological evaluations owing to ill health. Another participant in the training group was very slow at completing the paper-and-pencil FSNC task compared with performance expected from the performance simple arithmetic pretest measures (which means that participants were not doing the training tasks earnestly at all). The criteria are that speed of the paper-and-pencil training tasks in the first day of training was less than 80% of speed of the pretest arithmetic measure. These three participants were excluded from further analysis, leaving 17 participants in the FSNC group and 20 participants in the no-intervention group. Furthermore, two participants (one in the FSNC group and the other in the no-intervention group) who misunderstood the rules of the cognitive tasks used as outcome measures (characterized by few answers for simple tasks or chance-level accuracy) were excluded from the analyses involving those tasks.

### 2.2. Procedure

The FSNC training program comprised computerized Borland C++ programs developed in-house, which consisted of adaptive training of FSNC tasks. The tasks included three tasks that were performed using computer keys and three tasks that were performed using paper and a pencil. Participants in the training group underwent 5 days of training (approximately 4 h/day) within a 6-day period in the laboratory. The training on one day finished after the completion of a certain amount of training tasks, and the participants were allowed to take breaks when they thought that they needed breaks. Instructions and brief practice of all training tasks were given on the first training day before training began. All participants underwent MRI scanning and psychological tests immediately before and after this 6-day period. In other words, pretraining MRI scans and psychological tests were performed on day 1, training was provided from day 2 to day 7, and posttraining MRI scans and psychological tests were performed on day 8. The no-intervention group did not receive any training or perform any specific activity during the period separating the two MRI sessions. The timing and order of psychological tests and MRI sessions differed among participants (independent of the training group allocations) because, for MRI scans, only one participant could be scanned at a time and the psychological tests were performed in a group setting. Participants completed psychological test sessions when they were not participating in MRI scans. Because participants in the intervention group were required to participate in the 5-day training session during the 6-day intervention period, they had to complete the postexperiment evaluations 1 or 2 days after intervention completion.

The lack of an active control group (placebo training) has been common to almost all of the imaging studies of cognitive training. In particular, the use of the no-intervention group as a control group has been well described [10, 14–19]. We believe that it is appropriate and congruent with the custom of the research field. For details related to this discussion, please refer to our previous study [15].

### 2.3. Training Tasks

All three computerized tasks were adaptive tasks, in which the problems were presented for a fixed period of time that was adjusted based on a subject's performance (for details, see below). In all three computerized tasks, operations (simple calculations using a pair of single digits (e.g., 8–4)) were presented successively. The three computerized tasks included simple addition, subtraction, and multiplication (Figure 1). In these tasks, three simple calculations were presented in a vertical order (e.g., 6 + 7, 7 + 5, and 2 + 2) for each trial. The participants had to press the keys that corresponded to the digits of the answer to the first (top) of the three problems presented (in case of 6 + 7, the key to be pressed was 3, “tens” did not need to be keyed in response) before the next stimuli were presented (next trial). Participants could push the button whenever they wanted, but the button pushed at last during each trial was used to judge the correctness of answers. The keys 1, 2, 3, 4, 7, 8, 9, and 0 were pressed for answers of 1, 2, 3, 4, 7, 8, 9, and 0, respectively. However, in case of 5 and 6, the subject had to press the R and Y keys using their thumbs, respectively, so participants could push 10 buttons using 10 fingers. We instructed participants to press 10 keys using 10 fingers (i.e., the fifth finger of the left hand was used to press key 1, and the fourth finger of the left hand was used to press key 2; and the fifth finger of the right hand was used to press key 0). However, when it was impossible for the participants to adhere strictly to these instructions, they were allowed to press the keys with any finger. In the next trial, the second problem was presented at the top of the list, which was reordered so that the second highest problem was the one which was at the bottom of the list in the previous trial and a new problem appeared at the bottom of the new list (e.g., 7 + 5, 2 + 2, and 4 + 8 for the above example). A fixation was not used between the trials. The list of problems remained on the screen until the next trial started. This presentation enabled participants to solve the upcoming problem before the next trial (such as in the case of paper-and-pencil tasks) and created a kind of multitasking situation (such as in the case of paper-and-pencil tasks). In problems involving subtraction, the digits were presented such that the answers to the problem were not below 0. In these computerized tasks, performance of each block (a period during which operations were presented sequentially) was defined by the number of correct responses and each block ended after 24 trials. One session of each computerized task ended after 30 blocks with the exception of the first training day; on the first training day, one session of each computerized task ended after 20 blocks.

In all three computerized tasks, the difficulty (stimulus presentation rate) was modulated based on subject performance (the number of trials in which participants were able to input the correct answers (out of 24 trials) in one block is represented as *X* below) by multiplying by 0.99 or 100/99; that is, the participants' performance on each task was expressed as *X* in a certain block and the stimulus presentation rate as *A* in that block. When *X* was 0–6, in the next block, the stimulus presentation rate was *A*(0.99)^4^; when *X* was 7–9, in the next block, the stimulus presentation rate was *A*(0.99)^10−*X*^; when *X* was 10–12, in the next block, the stimulus presentation rate did not change; and when *X* was 13–24, in the next block, the stimulus presentation rate was *A*(100/99)^*X*−12^. For example, when participants answered correctly in 16 out of 24 trials in one block and the stimulus presentation rate of the block was 2 stimuli/s, then in the next trial, the stimuli presentation rate became 2*∗*(100/99)^18−12^≒2.1243 stimuli/s. Basically, with this procedure, when participants performed the tasks properly at a given speed, then in the next block, the stimulus presentation rate was increased based on how well the participants could perform the tasks properly. When participants could not perform the tasks properly at a given speed, then in the next block, the stimulus presentation rate was decreased based on their performance. When participants' performance was not so bad, then in the next block, the stimulus presentation rate did not change. In the computerized tasks, the participants began the training each day at the same level that they had finished at for each task on the previous day. The initial stimulus presentation rate was 1 stimulus/s. As for the difference in how many times participants met each operation (e.g., 1 + 4), for example, in the case of the computerized addition task, there were 64 possible operations (1 and 0 were removed from the problems and 8 × 8 = 64 operations existed) that occurred by the same possibility (repeat of the same operations could happen with a probability of 1/64 × 100 (%)). Also, as described above, participants faced addition operations 3600 times in the computerized addition task during the training period (24 trials (operations) × 30 blocks × 3 sessions × 1 day (first training day) + 24 trials (operations) × 30 blocks × 3 sessions × 4 days (second to fifth training days)). Thus, participants were expected to face each stimulus 56.25 times (3600/64) on average during the training period. Although the actual number must have differed among different operations due to the computerized randomization, due to the law of large numbers, that was not a significant concern.

In all three paper-and-pencil tasks, rows of problems of simple numerical calculations involving a pair of single digits (e.g., 8 − 4) were printed. These tasks involved simple addition, subtraction, and multiplication (Figure 1). Participants had to solve these problems from the top order and write down the answers (if the answers were two-digit numbers, they also had to write down the tens digits). The participants were instructed to answer as many questions as possible in 1 min. They had to perform this task 10 times/session.

Computerized and paper-and-pencil tasks were alternated, and when the participants had completed three sessions for each of these tasks, training for that day was declared to be complete. The order of these tasks were fixed and were as follows: (1) the paper-and-pencil addition task (1 min × 10 times); (2) the computerized addition task (30 blocks (1 block consists of 24 trials), only in the first training day, 20 blocks); (3) the paper-and-pencil multiplication task (1 min × 10 times); (4) the computerized multiplication task (30 blocks (1 block consists of 24 trials), only in the first training day, 20 blocks); (5) the paper-and-pencil subtraction task (1 min × 10 times); and (6) the computerized subtraction (30 blocks (1 block consists of 24 trials), only in the first training day, 20 blocks). These processes (1 through 6) were repeated three times each day.

Both computerized and paper-and-pencil tasks were included in training because increasing the variability of tasks, stimuli, and training context leads to more successful transfer of information [8, 20, 21].

### 2.4. Psychological Outcome Measures

For the evaluation of the pre- and posttraining effects on psychological measures, a battery of neuropsychological tests and questionnaires was administered. These cognitive tests were generally the same as in our previous study [22] and evaluated a wide range of cognitive functions, but the tests that showed low test-retest reliabilities or some other problems in our previous study were replaced by alternate tests.

#### 2.4.1. Arithmetic Tasks

[A] Arithmetic tasks, similar to the ones constructed by Grabner et al. [23], measured multiplication performance on two forms of one-digit × one-digit multiplication problems (a simple arithmetic task with numbers between 2 and 9) and two forms of two-digit × two-digit multiplication problems (a complex arithmetic task with numbers between 11 and 19). The two forms of each task were identical, but the numbers used in the problems were ordered differently. Each form of the simple and complex arithmetic tasks was presented with a time limit of 30 and 60 s, respectively.

#### 2.4.2. Nonverbal Reasoning Tasks

[B] These included the following: Raven's Advanced Progressive Matrices [12], a nonverbal reasoning task; [C] Cattell's Culture Fair Test [24], a nonverbal reasoning test.

#### 2.4.3. Working Memory Tasks

[D] These included the following: a (computerized) digit span task, a verbal working memory task (for the details of this task, see [25]); [E] a (computerized) visuospatial working memory task [10].

#### 2.4.4. Intelligence Test with Speeded Tasks

[F] The test used was the Tanaka B-type intelligence test [26]. Type 3B, which is for examinees in their 3rd year of junior high school and older, was used in this study. This task was mainly performed as previously described [10]. This test is a nonverbal mass intelligence test which does not include story problems but uses figures, single numbers, and letters as stimuli. In all subtests, participants had to complete as many problems as possible within a certain time (a few minutes). This test consists of a maze test (participants had to trace a maze with a pencil from start to finish), counting cubes (participants had to count the number of cubes piled up in three-dimensional ways), a displacement task (figures and numbers; participants had to substitute a figure (9 figures) with a number (1 to 9) according to a model chart), identification versus same-different judgments (Japanese kana characters; participants had to judge whether a pair of meaningless Japanese strings were the same), filling in a sequence of numbers (participants had to fill in the blanks of a number sequence with suitable numbers according to the rules of the number arrangement), marking figures (participants had to select forms which were identical to three samples from a series (sequence) of eight different forms), and filling in figures (participants had to complete uncompleted figures so that the uncompleted figures were the same as the sample figures when rotated).

#### 2.4.5. Simple Processing Speed Tasks and Executive Function (Inhibition Tasks)

[G] The task used was the Stroop task (Hakoda's version) [27], which measures response inhibition and impulsivity and which is the matching-type Stroop task. The following description is essentially the same as the description in our previous study [28]. Unlike the oral naming-type Stroop tasks, in the matching-type Stroop task (writing), participants had to choose and write down as many appropriate answers as possible from five options. This type of task enables the measurement of participants' performance correctly. The task consists of two control tasks (Word-Color task, Color-Word task), a reverse Stroop task, and a Stroop task. Reverse Stroop interference means the slowing of an output when participants have to provide the meaning of a word when there is a conflict between the meaning of the word and its printed color. In the Word-Color task, a color name (e.g., “blue”) is presented in the leftmost column. In addition, five columns are painted with five different colors and participants have to check the column whose color corresponds to the color name in the leftmost column. In the Color-Word task, the leftmost column is painted with a color, and five other columns contain color names. The participants have to check the column with the word corresponding to the name of the color painted in the leftmost column. In the reverse Stroop task, in the leftmost column, a color name is printed in another color (e.g., “blue” is printed in green) and five other columns are painted in five different colors. The participants have to check the column whose color corresponds to the color name in the leftmost column. In the Stroop task, in the leftmost column, a color name is printed in another color (e.g., “blue” is printed in green) and five other columns contain color names. The participants have to check the column with the word corresponding to the name of the color in which the word in the leftmost column is printed (Supplemental Figure 1 in Supplementary Material available online at http://dx.doi.org/10.1155/2016/5940634). During each task, the participants were instructed to complete as many tasks as possible in 1 min. Four tasks were performed in a fixed order, but the order of the task did not affect the performance of each task [27]. We used the Word-Color and Color-Word tasks as simple processing speed measures and Stroop and reverse Stroop tasks as inhibition measures [10].

#### 2.4.6. Creativity Task

[H] The S-A creativity test [29] is used to evaluate creativity through divergent thinking and involves three types of tasks (for details on the development of this instrument and its psychometric properties, refer to the technical manual for this test [29]). The first, second, and third tasks require participants to generate unique ways of using typical objects, imagine desirable functions for ordinary objects, and imagine the consequences of “unimaginable things” happening, respectively. The S-A test scores the four dimensions of the creative process (fluency, originality, elaboration, and flexibility). In this study, the sum of the graded scores for the four dimensions was used in the analysis. For more details including the psychometric properties of this test, sample answers to the questionnaire, and the manner in which they were scored, refer to our previous studies [30, 31].

Other than these cognitive tests, we collected several questionnaires designed to assess the traits or states of the participants, but these are not reported here. In most cases, these were self-report questionnaires evaluating participant behavior in daily life. They were designed to assess the traits of the participants and not the effect of the five-day intervention. Other than the self-report questionnaires, all neuropsychological assessments were performed by postgraduate and undergraduate students blinded to the group membership of the participants.

### 2.5. Group-Level Statistical Analysis of Behavioral Data

Behavioral data were analyzed using SPSS 16.0 (SPSS Inc., Chicago, IL, USA). Effects of FSNC training on each measure were analyzed by comparing the FSNC training group and no-intervention group using one-way analyses of covariance (ANCOVAs). In these ANCOVAs, the differences between pre- and posttest measures were computed by subtracting the preintervention value from the postintervention value and were entered as dependent variables. Also, in ANCOVAs of the psychological measures, the pretest scores were entered as covariates to exclude the possibility that any preexisting differences between groups in the measures would affect the results of each measure (see below for the covariates of imaging data analyses). Repeated measure analyses of variance (ANOVAs) have no superiority over this design as far as we know, but an obvious inferiority of ANCOVA is that repeated measures ANOVAs cannot correct the effects of pretest scores and thus cannot control the preexisting difference in the measures between the groups. Because the superiority (or beneficial effects) of intervention training was our primary interest, in our behavioral analysis, test-retest changes in the group of interest were compared to those in the control group using one-tailed tests (*P* < 0.05) [32, 33]. However, for psychological outcome measures in which “superiority” (or beneficial effects) was unclear (such as when the creativity test score was associated with an impaired selective attention system, psychosis, or cognitive disinhibition), two-tailed tests were used (for details, see [25, 34]).

We reported results that were only significant at the level of uncorrected data for multiple comparisons. This was partly because this study's investigation of cognitive functions had an exploratory nature (administration of a wide range of cognitive tests of major cognitive functions regardless of the existence of strong a priori hypotheses). This was also partly because we followed the customs of the field in not performing a correction for multiple comparisons [10, 22, 32, 33, 35–41]. However, we have also reported the statistical results after correction for multiple comparisons. The correction for multiple comparisons was performed using the false discovery rate (FDR) and the graphically sharpened method [42]. FDR was applied to analyses of 12 tests that are presented in Table 1.

#### 2.5.1. Confirmation of the Significant Behavioral Findings Using the Data of the Active Control Group in the Previous Study

However, this study had a smaller sample size than some previous studies [43] involving this type of behavioral analysis. Furthermore, it involved a number of cognitive tasks. Moreover, FSNC training led to significant improvements in only one of two measures each for processing speed and executive functioning in the main analysis (see Results). We therefore performed an additional analysis that included data from the active control (placebo training) group of our previous study [22] to increase the statistical power and reliability of the present study results and to directly address the lack of effect in the placebo training group.

In this previous study [22], the pre- and posttest measures were also separated by 1 week, and several (but not all) of the cognitive tests were performed in the same manner as in the present study, with participants having similar characteristics (healthy young adults). Furthermore, the placebo training group in this previous study [22] had the same training period, training time, and training frequency as the training group in the present study. This previous study had a no-intervention control group in addition to the active control group, but when the active control group was compared with the no-intervention group, no effects of placebo training were observed on task performance [22]. Thus, please note, in the description of the present paper, that there are 3 control groups (one no-intervention group in the experiment of the present study, one active control group from the previous study, and one no-intervention group from the previous study). And the data from the no-intervention group in the experiment of the present study and the active control group from the previous study was used in the confirmatory analysis of this subsection (further addition of the data of no-intervention group from the previous study just strengthened the *P* values of the significant results in the confirmatory analysis of this subsection).

### 2.6. Image Acquisition

All MRI data acquisition was performed using a 3-T Philips Achieva scanner. Using a MPRAGE sequence, high-resolution *T*1-weighted structural images (240 × 240 matrix, TR = 6.5 ms, TE = 3 ms, FOV = 24 cm, 162 slices, slice thickness = 1 mm) were acquired. Arterial Spin Labeling (ASL) was performed to measure resting CBF. It was performed with quantitative signal-intensity targeting by alternating the radio-frequency pulse labeling of arterial regions (QUASAR), a pulsed ASL method [44]. Details of the sequence and the method for calculating perfusion parameters have been outlined elsewhere [44–46]. The actual imaging parameters were as follows: 64 × 64 matrix, TR = 300 ms, TE = 22 ms, FOV = 24 cm, 7 slices, slice thickness = 7 mm (2.0 mm gap), SENSE = 2.5, 84 averages, and scan duration = 5 min 52 s. We determined the position of the slice by putting the fourth of seven slices on the body of the corpus callosum in the coronal scout view [47]. During ASL scan, the participants were instructed to remain still with their eyes closed, as motionless as possible, and not to sleep or think about anything in particular.

## 3. Preprocessing and Analysis of Structural Data

VBM, a method of* in vivo* study of human brain structures that can detect changes in rGM caused by training [17, 48], was used to investigate the effect of FSNC training on brain structures. Morphological data were preprocessed using the default cross-sectional methods of VBM2 software [49] and as performed in our previous study [22] as an extension of SPM2. We used cross-sectional methods with VBM2 software, and pre- and postimages of each participant were preprocessed independently to avoid asymmetry-induced bias [50]. To reduce the scanner-specific bias, we used a customized gray matter (GM) anatomical template and prior probability maps of GM and white matter images created from *T*1-weighted structural images obtained using this scanner in our previous study [30, 51]. Next, the *T*1-weighted structural images from each subject were segmented into GM and white matter partitions using the abovementioned custom GM and white matter prior probability maps. The resulting images included extracted GM and white matter partitions in the native space. The GM partition was then normalized to the abovementioned custom GM probability map. The normalization parameters determined from this initial step were applied to the native *T*1-weighted structural image. These normalized *T*1-weighted structural data were then segmented into GM and white matter partitions. In addition, we performed a volume change correction (modulation) by modulating each voxel with the Jacobian determinants derived from spatial normalization, allowing the determination of regional differences in the absolute amount of GM [52]. Subsequently, all images were smoothed by convolving them with an isotropic Gaussian Kernel of 12 mm full-width at half maximum (FWHM). 12-mm FWHM smoothing value is warranted in the cluster size test for VBM (see the paragraph below).

VBM2 was used instead of VBM5 or VBM8 for the preprocessing of *T*1-weighted structural imaging data because *T*1WIs obtained using our MPRAGE sequence (see above) were incompatible with preprocessing with VBM5/SPM5 and VBM8/SPM8. When VBM5 or SPM5 was used, many apparent segmentation errors occurred, unlike when the optimized protocol of VBM2 was used. Segmentation errors apparent at first glance were not found when VBM2 or VBM8 was used. However, when VBM8 was used, the test-retest reliability of total GMV of 50 participants who participated in a 1-week longitudinal intervention study in which *T*1WI was taken on the first day of the experiment and 1 week thereafter [10] was 0.746, whereas when VBM2 was used, the reliability was 0.980. It should be noted that this longitudinal intervention experiment is the same as the experiment in this study. In this experiment, 58 participants in three groups (FSNC training, no-intervention group, and PS-training group) completed the longitudinal experiment properly and enrolled in the analysis. Among these participants, the data from 50 participants were used to calculate reliability. Visual inspection was also conducted on the results of segmentation as a quality check. The results indicated that the quality did not seemingly differ between the pre- and postexperiment results for segmentation. These procedures (preprocessing with VBM2 and statistical analyses using different versions of SPM/VBM) were also used in previous studies [17, 22, 30, 51]. Although this data does not indicate that preprocessing with VBM5/VBM8 is worse, it does say something about the compatibility between *T*1WIs of certain sequences and VBM5/VBM8. For more extensive discussions related to this issue, please refer to [53].

In the group-level analysis, we tested for a change in rGMV between the first and second time points by comparing the training and control groups (i.e., group × time interaction). The statistical significance level was set at *P* < 0.05, corrected for multiple comparison (FWE) at the nonisotropic adjusted cluster level [54] with an underlying voxel-level of *P* < 0.0025. Nonisotropic adjusted cluster size tests can and should be applied when cluster size tests are applied to nonstationary data (i.e., are not uniformly smooth), such as VBM data [54]. In this nonisotropic cluster size test of random field theory, a relatively higher cluster-determining voxel-level threshold combined with high smoothing values of more than six voxels leads to appropriate conservativeness in real data. With high smoothing values, an uncorrected threshold of *P* < 0.01 seems to lead to too many false positives, whereas that of *P* < 0.001 seems to lead to slight conservativeness [55].

Furthermore, we investigated whether preexisting differences in rGMV (in the preintervention scan) existed between the training and control groups at the whole brain level using ANOVA. In all of these group analyses of morphological data, we included only voxels with a GM value >0.10 to avoid the possibility of partial volume effects around the borders between GM and WM as well as those between GM and the cerebrospinal fluid (CSF).

We did not control the global signals across all brain imaging analyses, as was the case for almost all intervention imaging studies.

## 4. Preprocessing and Statistical Analysis of Resting rCBF Data

Maps of raw resting rCBF and the longitudinal relaxivity (*R*1 = 1/*T*1) of each subject were obtained using dedicated software running on IDL (Research Systems, Boulder, Colorado) ([44]; National Neuroscience Institute, Singapore). The following constants were used in CBF calculation: *T*1 of arterial blood, 1.65 s; inversion efficiency, 95%; blood-brain partition coefficients for GM and WM (0.98 and 0.82, resp.) [44].

Preprocessing and data analysis were performed using SPM5 implemented in Matlab, except in the segmentation procedure (see below), where SPM2 was used. This approach was used because the *T*1-weighted images acquired using the MPRAGE sequence were incompatible with VBM5 and SPM5 preprocessing and resulted in numerous apparent segmentation errors.

We segmented the *T*1-weighted images into GM, WM, and CSF. Then, using the segmented GM and WM images as masks, we removed the parts that did not belong to the GM and WM images from the *T*1-weighted image and created images that solely consisted of GM and WM (designated “GM + WM *T*1-weighted image”).

*R*1 maps from the pre- and post-MRI scans of each subject, which lack the skull and skin section of the head and retain their alignment with the rCBF maps of each subject, were coregistered to the GM + WM *T*1-weighted image from the pre-MRI scan of each subject using the within-subject registration method. Asymmetry-induced bias was avoided during preprocessing of ASL images using the third structural image for the registration of pre- and post-ASL images (although this image is taken before the experiment, since the structural image and ASL images were collected separately, this fact does not cause asymmetry-induced bias).

The raw *T*1-weighted structural image from the pre-MRI scan of each subject, which maintained its alignment with the GM + WM *T*1-weighted image from the pre-MRI scan and rCBF maps from the pre- and post-MRI scans, was then normalized to our original template of the *T*1-weighted structural image which was established in our previous study on images of young adults taken with our scanner [56].

Using the parameters for this normalizing procedure, rCBF maps from the pre- and post-MRI scans of each subject were spatially normalized to create images with 2 × 2 × 2 mm^3^ voxels. The processed normalized rCBF maps from the pre-and post-MRI scans were then spatially smoothed using a Gaussian kernel of 12 mm FWHM. Finally, the signal change in resting rCBF between the pre- and post-scan images was computed at each voxel by subtracting the former image from the latter for each subject. The maps representing resting rCBF from the pre-MRI scan and those representing changes in resting rCBF from the pre-MRI scan to the post-MRI scan were subjected to the group-level analysis (see below). The total CBF in the whole brain may change through these processes, as was the case with when total signals in BOLD signal images and FA images change through normalization procedures [10, 57], it did not cause problems in this study.

In the group-level imaging analysis, we tested for group-wise differences in changes in resting rCBF. We performed voxel-wise ANCOVAs using the differences in each measure between the pre- and post-MRI scan values at each voxel as dependent variables and the pre-MRI scan values at each voxel as independent variables. These voxel-wise ANCOVAs were performed using Biological Parametrical Mapping (BPM) [58], which is implemented in SPM5 and using images representing prescan resting rCBF and pre/postscan changes in resting rCBF. This analysis using BPM was not applied to rGMV analysis because BPM does not handle the nonisotropic adjusted cluster size test, which was used in the rGMV analysis.

Regions of significance for the ASL analysis were inferred using cluster-level statistics of the standard SPM method [59]. Only clusters with *P* < 0.05, after correction for multiple comparisons (FWE) at cluster size with a voxel-level cluster-determining threshold of *P* < 0.0025, uncorrected, were considered statistically significant in this analysis.

## 5. Investigation of Associations between Performance Changes of Training Tasks and Neural Changes

We next investigated whether there was an association between changes in performance on training tasks and neural changes where the effects of FSNC training were observed through simple regression analyses. Individual performance change was calculated as follows. First, each task's best performance (in computerized tasks: the shortest interstimulus interval (ISI) of blocks in which participants answered correctly in more than half of the trials; in the paper-and-pencil tasks: the largest number of items completed in a single trial) of the first training day and that of the last training day were calculated across all six training tasks. Then, the ratio of change in these performances was calculated. Finally, the mean ratio of the three computerized tasks was averaged and that of three paper-and-pencil tasks was averaged and evaluated as the degree of the performance increase during the course of training. Next, we extracted the mean value of the pre- to posttraining changes in rGMV or resting rCBF in each of the significant clusters identified above. Then, simple regression analyses were performed to determine the association between the improvements in performance (either computerized tasks or paper-and-pencil tasks) on FSNC training tasks, and the neural changes of each cluster were calculated as described above. We employed one-tailed analyses to investigate the association between the increases in performance on FSNC training tasks and mean changes in each cluster in directions in which FSNC training effects were seen because that was our sole hypothesis and interest. In other words, when FSNC training resulted in a decrease in neural values, the associations between individual task performance increase and mean decrease in neural values for the cluster were investigated and vice versa.

## 6. Results 

### 6.1. Training Data

**Figure 2:**
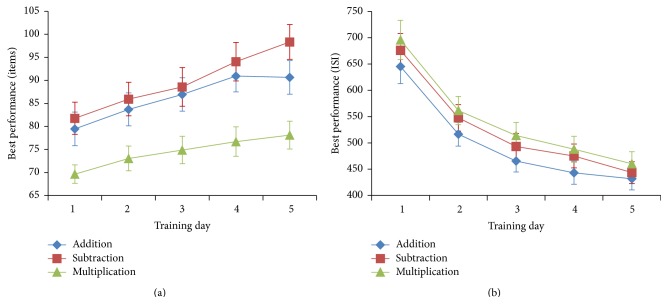
Practice-related performance increase in (a) paper-and-pencil tasks and (b) computerized tasks after FSNC training. Both paper-and-pencil and computerized tasks consisted of addition, subtraction, and multiplication tasks. Practice resulted in a significant increase in performance across all training tasks (in paper-and-pencil tasks, performance was measured in terms of the largest number of items answered in one trial; in the computerized tasks, performance was measured in terms of the shortest interstimulus interval (ISI) of blocks in which participants answered correctly in more than half of trials) from the first to the last day of training (one-tailed paired *t* test, *P* < 0.001). Error bars represent standard errors.

Practice resulted in a significant increase in performance across all six training tasks (in computerized tasks: the shortest interstimulus interval (ISI) of blocks, in which participants answered correctly in more than half of trials in the day, was decreased; in the paper-and-pencil tasks: the number of items completed in a single trial was increased) from the first to the last day of training (paired *t*-test, *P* < 0.001 for all six training tasks; Figure 2).

### 6.2. Effect of FSNC Training on Psychological Outcome Measures

Compared with the control group, the training group showed significantly larger pre- to posttest increases in performance on a simple arithmetic (multiplication) task (*P* < 0.001, uncorrected), a complex arithmetic (multiplication) task (*P* = 0.034, uncorrected), a processing speed measure (Color-Word task, *P* = 0.005, uncorrected), and an executive function task (reverse Stroop task, *P* = 0.030, uncorrected; Table 1).

These results revealed that FSNC training improved performance on an untrained complex arithmetic task, a simple processing speed task, and a speeded executive function task. However, FSNC training consistently failed to improve performance on tasks involving working memory, nonverbal reasoning, and creativity measures.

We reported results with significant values that were uncorrected for multiple comparisons for the reasons described in Methods. However, even when correction for multiple comparisons was performed using FDR, the results of a simple arithmetic (multiplication) task and a processing speed measure (Color-Word task) remained significant (*P* = 0.003, corrected and *P* = 0.018, corrected, resp.), and the results of a complex arithmetic (multiplication) task and an executive function task (reverse Stroop task) still showed a nearly significant tendency (*P* = 0.060, corrected and *P* = 0.060, corrected, resp.).

#### 6.2.1. Confirmation of the Significant Behavioral Findings

The mean ± SD of the scores of the tests for the active control group from the previous study are reproduced in Supplemental Table 1.

In the present analysis, comparison of the combined control group (the data from the no-intervention group in the experiment of the present study and the active control group from the previous study) and the FSNC training group showed that all significant results in the first behavioral analysis remained significant (simple arithmetic task, *P* < 0.001, uncorrected; complex arithmetic task, *P* = 0.034, uncorrected; Color-Word task, *P* = 0.018, uncorrected; reverse Stroop task, *P* = 0.009, uncorrected). Moreover, in this second behavioral analysis, the FSNC training group showed tendency of pre- to posttest increases in performance on the Stroop task (*P* = 0.061, uncorrected). No other changes were observed in the significance of the other tests (RAPM and a creativity measure) used in our previous study [22]. When the correction for multiple comparisons using FDR that was described above was performed against these *P* values for the comparisons between the FSNC training group and the two control groups in the cases where data from the two control groups were available, in addition to the *P* values for the comparisons between the FSNC training group and one control group in the present experiment in the cases where data from the previous study was not available, three of the significant uncorrected results (uncorrected) in this subsection (simple arithmetic task, Color-Word task, and reverse Stroop task) remained significant even after the correction of multiple comparisons (see Supplemental Table 1) and the result of the complex arithmetic task was also close to significance (*P* = 0.059, corrected). The results of this comparison further support the significance of the conclusions drawn in this study.

### 6.3. Effect of FSNC Training on GM Structures

**Figure 3:**
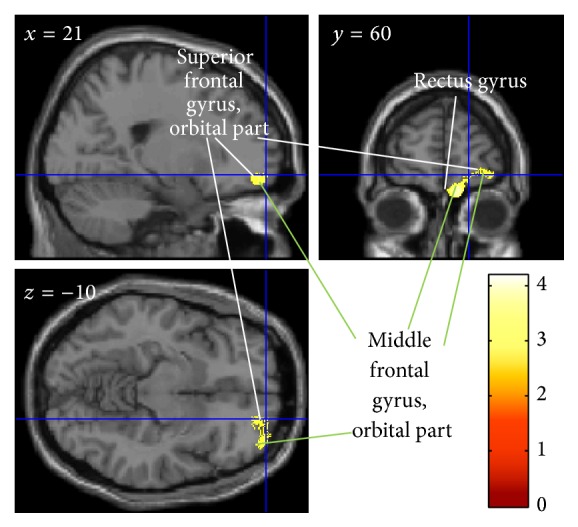
Effect of FSNC training on rGMV. The results are shown with *P* < 0.0025, uncorrected. Compared with the control group (no-intervention), the FSNC training group showed a significant decrease in rGMV in the right frontopolar area.

VBM analysis tested for a change in brain structure after the intervention by comparing pre- and posttest images from the training and control groups (group × time interaction). This analysis revealed that FSNC training resulted in a statistically significant greater decrease in rGMV around the right frontopolar area (the right middle and superior frontal gyri; Figure 3). No statistically significant FSNC training-related increases in rGMV were observed. For statistical values, see Table 2.

Furthermore, whole-brain ANOVA showed no significant regional differences in rGMV between the training and control groups before the intervention (pre-MRI scan; *P* > 0.2, corrected for multiple comparisons).

### 6.4. Effect of FSNC Training on Resting rCBF

**Figure 4:**
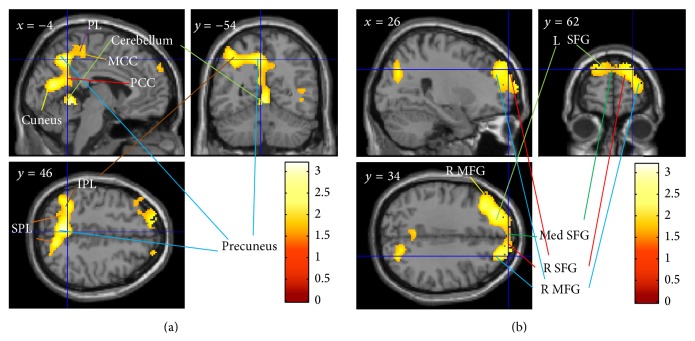
Effect of FSNC training on resting rCBF. The results are shown with *P* < 0.05, corrected for multiple comparisons at cluster size, with an underlying voxel-level of *P* < 0.05, uncorrected. FSNC training resulted in an increase in resting rCBF in an extended anatomical cluster in the posterior region (a) and in an extended anatomical cluster primarily located in the bilateral frontopolar areas (b). MCC: middle cingulate gyrus. PCC: posterior cingulate gyrus. IPL: inferior parietal lobule. SPL: superior parietal lobule. SFG: superior frontal gyrus. MFG: middle frontal gyrus. Med SFG: medial part of the superior frontal gyrus. PL: paracentral lobule.

We next compared changes in resting rCBF in the training and control groups. However, no region showed statistically significant changes at a threshold of *P* < 0.05, corrected for multiple comparisons at the cluster level with a cluster-determining voxel-level threshold of *P* < 0.0025, uncorrected. ASL, which was used to measure resting rCBF, may lack regional sensitivity due to a number of reasons [56]. Thus, we performed statistical analysis with a more lenient cluster-determining voxel-level threshold (*P* < 0.05, uncorrected) to determine whether any weak but widely significant results could be observed. This analysis revealed a statistically significant FSNC training-related (training group versus control group) increase in resting rCBF in (a) a large cluster that included the precuneus, left postcentral gyrus, right middle temporal gyrus, left inferior parietal lobule, right superior occipital lobe, paracentral lobule, posterior cingulate cortex, right parahippocampal gyrus, and left superior parietal lobule (Figure 4(a)) and (b) a large cluster mainly in the bilateral frontopolar areas but also in the bilateral middle and superior frontal gyri (Figure 4(b)). No regions showed statistically significant FSNC training-related decreases in resting rCBF. The frontal cluster located close to the significant cluster of FSNC-related rGMV change but did not overlap when the latter cluster was formed with a threshold of uncorrected *P* < 0.0025 but rather overlapped when the latter cluster was formed with a threshold of uncorrected *P* < 0.01, a more lenient threshold. Because much of the orbitofrontal area was barely included in analyses of ASL due to the limited scan area of this scan method (for areas of ASL analyses, see supplemental Figure 2), it is difficult to conclude something regarding the overlap of the cluster of rGMV and cluster of ASL in the frontal area. For statistical values, see Table 3.

### 6.5. Associations between Neural Changes and FSNC Training Tasks' Performance Changes

Simple regression analyses that tested correlations between improvements in the performance of computerized FSNC training tasks and paper-and-pencil FSNC training tasks and the amount of rGMV and resting rCBF changes in the significant clusters identified in this study's analyses (see above) were also performed (2*∗*2 = 4 analyses). The results only revealed the tendency of the negative correlation between rGMV change in the cluster of the right OFC and computerized FSNC tasks' performance change (*P* = 0.08, *t* = −1.45). The result may suggest an association between an rGMV decrease on the right OFC and an increase in FSNC training tasks performance; however, due to the nonsignificant tendency of the results, we cannot draw conclusions.

## 7. Discussion

To the best of our knowledge, the present study is the first to reveal the effects of FSNC training on cognitive function, rGMV, and resting rCBF in healthy young adults. Our previous study showed that cognitive training including FSNC improves performance on untrained processing speed and speeded executive functioning tasks [4] in the elderly. In this study, consistent with our previous study, FSNC training was associated with improvements in performance on simple processing speed, speeded executive functioning, and simple and complex arithmetic tasks. Moreover, consistent with our hypothesis proposing the involvement of the prefrontal cortex, FSNC training was associated with a reduction in rGMV and an increase in resting rCBF in the prefrontal cortex, specifically in the frontopolar areas. FSNC training was also associated with a weak but widespread increase in resting rCBF in an anatomical cluster in the posterior region.

The FSNC-related change in rGMV in the right frontopolar area (Figure 3) may be caused by a requirement for a certain type of multitasking operation during FSNC and may mediate the FSNC-related improvement in performance on untrained cognitive tasks. The frontopolar region is said to be involved with multitasking, and lesions in this region lead to impaired multitasking [60]. One of the important cognitive operations involved in faster performance on the FSNC task is observing and solving the next problem whilst writing down the answer to the current problem (a certain form of multitasking is apparently required here; Figure 1). We specifically designed the computerized tasks to have the same characteristics as the pen-and-paper task, where participants could look ahead to the next problem. This multitasking-like cognitive operation may require the frontopolar area, and FSNC training may lead to a change in rGMV in this region. This cognitive operation appears to be required for a number of cognitive tasks in which participants have to solve as many problems as possible. Thus, a certain type of multitasking operation in FSNC training may affect the right frontopolar area, which in turn may affect other types of cognitive tasks that require the same cognitive operation. Please note that the present cognitive training has few commonalities with the working memory training using calculations used in our previous study (for details, see [22]), and a lack of regional overlap in the effects of cognitive training is expected.

On the other hand, it has been proposed that there are two networks for calculation in the brain (one for exact calculation and the other for approximate calculation) [6]. The former network involves the frontopolar area as well as the posterior parietal region, which also showed significant neural changes in this study. Although, in the previous study, this activation was found in the left hemisphere, in this study, a similar tendency was also seen in the left hemisphere. Also, a closer look at the clusters of significant effects for the two studies in this area revealed a substantial overlap. Thus, as the present study involved training for exact number calculation, the changes in neural systems in this study may be regarded as changes in neural systems involved in exact calculation.

Decreases in rGMV observed after just 1 week of intensive (such as four hours per day) cognitive training are consistent with those observed in two of our previous studies of cognitive training [10, 22]. Decrease of rGMV in some regions after a short period of the intervention was seen in a number of other previous studies [48, 61, 62]. In our previous studies [10, 22], we suggested that cognitive training may lead to nonlinear changes (an initial increase followed by a decrease) in rGMV and that these changes are affected by training length and intensity (greater intensity leads to a more rapid nonlinear change) [10]. This suggestion was based on the results of previous studies as well as on a review of previous studies. Further, the increase of rGMV, which is often seen in longitudinal studies (e.g., [63]), was suggested to correspond to an initial increase in this process. We regarded usage-dependent selective elimination of synapses [64], which underlies day-to-day experience-dependent neural plasticity [65], as a potential mechanism underlying the decrease in rGMV. These notions may be consistent with the recent findings that learning new processes can lead to a transient increase of spine formation and that this rapid spinogenesis is followed by an enhanced elimination of spines that existed before training [66].

FSNC training may increase resting rCBF in the frontopolar and posterior regions through changes in the capillary network and increases in metabolic demand. The posterior parietal, posterior temporal, and occipital regions are recruited during simple numerical calculation [5]. In particular, previously known arithmetical facts appear to be accessed from the memory via the angular gyrus, while the intraparietal sulci are involved in tasks involving explicit representation of magnitude, such as subtraction [67]. Thus, together with the frontopolar area, the present results relating to resting rCBF may show experience-dependent plasticity of resting rCBF in performance on FSNC tasks. Experience-dependent neural plasticity involves increases in the width and density of capillaries [68, 69] and mitochondria [68], which lead to increased metabolic demand. FSNC training may increase resting rCBF through such changes. Alternatively, the increase of rCBF may just reflect prolonged enhancement of the default activity in these regions. These activity changes as well as changes of synapse and spines, genesis of cells, and angiogenesis occur within days to weeks [70]. Thus, it is unsurprising that there can be neural changes after only 1 week of intervention.

Consistent with our hypothesis, FSNC training led to improved performance on executive function (reverse Stroop task), processing speed, and simple and complex arithmetic tasks. Our previous study showed that a brain training game including FSNC improved executive function and processing speed [4]. This study further supports the notion that training involving complex speeded tasks leads to improvements in processing speed and certain types of executive functioning (possibly in speeded tasks). Moreover, our study extended the previous findings and showed that FSNC training alone can improve processing speed and executive function in healthy young adults. Furthermore, this study is consistent with our previous study in that training did not lead to improved performance on working memory measures [4]. FSNC training also did not improve performance on nonverbal reasoning and creativity tasks. If anything, a trend for FSNC training to decrease performance on creativity tasks was observed. Thus, these findings appear to show that this type of training does not lead to improvements in nonverbal reasoning, working memory, or creativity.

This study has the same limitations as our previous studies of cognitive training [10, 15, 19, 22]. The multiple training programs used in this study (computerized and paper-and-pencil tasks and three different types of operations) and previous studies ([33], e.g., [71]) are believed to strengthen transfer effects [8, 9]. They may also make it difficult to observe the effects of each training program individually [72]. The next limitation of this study is the complex training protocols [37, 43], which are commonly observed in this kind of study whether the training is about working memory training [43], video gaming [73], or meditation [37]. They typically have none of the strict control groups or conditions included in normal fMRI studies. Thus, the present study did not and did not even attempt to separate the effects of motor components, attention components, speed components, and components of some types of multitasking as described above from the effects of FSNC and we regard them essential and inseparable components of FSNC whenever the FSNC is performed. Furthermore, we used a lenient voxel-determining threshold in the rCBF analysis because ASL may lack in regional sensitivity due to many reasons. However, weak but widespread effects were still observed. The cluster-level statistic can control for type I error when the cluster-determining voxel-level threshold is lenient [74]. However, because of the nature of this statistical method, when a lenient cluster-determining voxel-level threshold is applied, the results cannot specify the exact location of the regions that have effects of interest [59]. Thus, the exact locations in which FSNC training has an effect on resting rCBF remain to be verified.

The executive function and frontopolar areas affected by FSNC training play important roles in higher order cognitive functioning in humans [60, 75]. Thus, the present findings have implications for plasticity of human higher-order cognitive functioning as well as implications for the application of FSNC training in fields such as education. Further, present results give new insights into the neural and the cognitive mechanisms, with which the FSNC training improves cognitive functions.

## Supplementary Material

Supplementary materials contain the table which described the data of the active control group in our previous study (Supplemental Table 1), the figure which showed the schema of the stroop tasks that we used (Supplemental Figure 1), and the figure which showed the areas of regional cerebral blood flow during rest (Supplemental figure 2).

## Figures and Tables

**Table 1:** Pre- and posttest scores for psychological measures (mean ± SEM).

	FSNC training		Control		Planned contrast in ANCOVA^c^	*P* value^c^ (uncorrected, corrected^d^)
	Pre	Post	Pre	Post		
Arithmetic						
Simple arithmetic (items)	30.7 ± 1.2	37.8 ± 1.5	33.0 ± 1.1	35.0 ± 1.3	FSNC training > control	4.28 *∗* 10^−4^, 0.003
Complex arithmetic (items)	7.79 ± 1.23	9.59 ± 1.71	7.03 ± 0.44	7.65 ± 0.52	FSNC training > control	0.0340, 0.060
Nonverbal reasoning						
RAPM^a^ (score)	27.7 ± 0.9	30.7 ± 0.9	28.1 ± 0.8	30.1 ± 0.9	FSNC training > control	0.112, 0.157
CCFT^b^ (score)	31.7 ± 1.1	32.8 ± 2.0	29.8 ± 1.0	35.0 ± 1.3	FSNC training > control	0.754, 0.440
Working memory (WM)						
Digit span (score)	37.9 ± 1.1	37.9 ± 1.2	36.5 ± 1.9	37.6 ± 1.7	FSNC training > control	0.702, 0.440
Visuospatial WM (score)	25.8 ± 1.7	28.6 ± 1.8	29.8 ± 1.2	30.6 ± 1.2	FSNC training > control	0.241, 0.211
Intelligence test with speeded tasks						
Tanaka B type intelligence test	114.1 ± 3.5	125.2 ± 3.4	118.7 ± 2.8	127.6 ± 2.8	FSNC training > control	0.301, 0.214
Simple processing speed						
Word-Color task (items)	74.2 ± 2.0	81.1 ± 1.4	73.8 ± 1.3	80.2 ± 1.2	FSNC training > control	0.306, 0.214
Color-Word task (items)	54.2 ± 1.8	59.6 ± 1.6	52.9 ± 1.4	55.3 ± 1.7	FSNC training > control	0.005, 0.018
Executive function (inhibition)						
Reverse Stroop task (items)	60.8 ± 2.3	68.0 ± 1.7	61.3 ± 1.6	66.0 ± 1.5	FSNC training > control	0.030, 0.060
Stroop task (items)	50.9 ± 2.1	55.0 ± 2.1	47.8 ± 1.6	51.0 ± 1.7	FSNC training > control	0.158, 0.158
Creativity						
S-A creativity test (total grade)	23.4 ± 2.0	23.2 ± 1.6	26.1 ± 1.4	27.0 ± 1.3	Two-tailed	0.152, 0.158

^a^Raven's Advanced Progressive Matrices.

^b^Cattell's Culture Fair Test.

^c^One-way ANCOVAs with test-retest differences in psychological measures as dependent variables and pretest scores on the psychological measures as covariates.

^d^ *P* values of results that were corrected for multiple comparisons using FDR.

**Table 2:** Brain regions with a significant greater decrease in rGMV in FSNC training.

Area		*x*	*y*	*z*	*T* score of the peak voxel	Corrected *P* value (cluster)	Raw cluster size (mm^3^)
Frontopolar area (superior frontal gyrus, orbital part/middle frontal gyrus, orbital part/superior frontal gyrus, and medial orbital/gyrus rectus)	R	13	60	−22	4.18	0.023	2128

No other significant results were observed.

**Table 3:** Statistical values of clusters with a greater increase in resting rCBF in FSNC training as well as their subpeaks.

Area		*x*	*y*	*z*	*T* score of the peak voxel	Corrected *P* value (cluster)	Raw cluster size (mm^3^)
Posterior cluster [angular gyrus (B)/calcarine cortex (B)/middle and posterior cingulate gyrus (B)/posterior cingulate gyrus (B)/cuneus (B)/lingual gyrus (B)/middle and superior occipital lobe (B)/paracentral lobule (L)/parahippocampal gyrus (R)/inferior parietal lobule (L)/superior parietal lobule (B)/postcentral gyrus (L)/precuneus (B)/inferior and middle temporal lobe (R)/cerebellum (B)]							
Cerebellum		2	50	−6	3.18	0.001	47512
Calcarine cortex	L	−10	−70	16	2.81		
Precuneus	L	−8	−58	46	2.75		
Anterior cluster [middle frontal gyrus (B)/superior frontal gyrus (B)/medial part of superior frontal gyrus (B)]							
Middle frontal gyrus	L	−26	48	36	2.78	0.011	31376
Middle frontal gyrus	L	−38	32	36	2.53		
Superior frontal gyrus	R	22	54	34	2.49		

No other significant results were observed.

## References

[1] LeAdelle P., McGrew K. S., Knopik S. N., Ford L. (2005). The general (g), broad, and narrow CHC stratum characteristics of the WJ III and WISC-III tests: a confirmatory cross-battery investigation. *School Psychology Quarterly*.

[2] Uchida S., Kawashima R. (2008). Reading and solving arithmetic problems improves cognitive functions of normal aged people: a randomized controlled study. *Age*.

[3] Kawashima R., Okita K., Yamazaki R. (2005). Reading aloud and arithmetic calculation improve frontal function of people with dementia. *Journals of Gerontology—Series A: Biological Sciences and Medical Sciences*.

[4] Nouchi R., Taki Y., Takeuchi H. (2012). Brain training game improves executive functions and processing speed in the elderly: a randomized controlled trial. *PLoS ONE*.

[5] Kawashima R., Taira M., Okita K. (2004). A functional MRI study of simple arithmetic—a comparison between children and adults. *Cognitive Brain Research*.

[6] Dehaene S., Spelke E., Pinel P., Stanescu R., Tsivkin S. (1999). Sources of mathematical thinking: behavioral and brain-imaging evidence. *Science*.

[7] Kennedy K. M., Raz N. (2009). Aging white matter and cognition: differential effects of regional variations in diffusion properties on memory, executive functions, and speed. *Neuropsychologia*.

[8] Sweller J., Van Merrienboer J. J. G., Paas F. G. W. C. (1998). Cognitive architecture and instructional design. *Educational Psychology Review*.

[9] Goldstone R. L. (1998). Perceptual learning. *Annual Review of Psychology*.

[10] Takeuchi H., Taki Y., Hashizume H. (2011). Effects of training of processing speed on neural systems. *The Journal of Neuroscience*.

[11] Oldfield R. C. (1971). The assessment and analysis of handedness: the Edinburgh inventory. *Neuropsychologia*.

[12] Raven J. (1998). *Manual for Raven's Progressive Matrices and Vocabulary Scales*.

[13] Snow R. E. (1989). Toward assessment of cognitive and conative structures in learning. *Educational Researcher*.

[14] Dahlin E., Neely A. S., Larsson A., Bäckman L., Nyberg L. (2008). Transfer of learning after updating training mediated by the striatum. *Science*.

[15] Takeuchi H., Taki Y., Nouchi R. (2013). Effects of working memory-training on functional connectivity and cerebral blood flow during rest. *Cortex*.

[16] Bäckman L., Nyberg L., Soveri A. (2011). Effects of working-memory training on striatal dopamine release. *Science*.

[17] Ilg R., Wohlschläger A. M., Gaser C. (2008). Gray matter increase induced by practice correlates with task-specific activation: a combined functional and morphometric magnetic resonance imaging study. *The Journal of Neuroscience*.

[18] Scholz J., Klein M. C., Behrens T. E. J., Johansen-Berg H. (2009). Training induces changes in white-matter architecture. *Nature Neuroscience*.

[19] Takeuchi H., Taki Y., Nouchi R. (2014). Effects of multitasking-training on gray matter structure and resting state neural mechanisms. *Human Brain Mapping*.

[20] Green C. S., Bavelier D. (2008). Exercising your brain: a review of human brain plasticity and training-induced learning. *Psychology and Aging*.

[21] Yamnill S., McLean G. N. (2001). Theories supporting transfer of training. *Human Resource Development Quarterly*.

[22] Takeuchi H., Taki Y., Sassa Y. (2011). Working memory training using mental calculation impacts regional gray matter of the frontal and parietal regions. *PLoS ONE*.

[23] Grabner R. H., Ansari D., Reishofer G., Stern E., Ebner F., Neuper C. (2007). Individual differences in mathematical competence predict parietal brain activation during mental calculation. *NeuroImage*.

[24] Cattell R. B., Cattell A. K. S. (1973). *Measuring Intelligence with the Culture Fair Tests*.

[25] Takeuchi H., Taki Y., Hashizume H. (2011). Failing to deactivate: the association between brain activity during a working memory task and creativity. *NeuroImage*.

[26] Tanaka K., Okamoto K., Tanaka H. (2003). *Manual of New Tanaka B Type Intelligence Test*.

[27] Hakoda Y., Sasaki M. (1990). Group version of the Stroop and reverse-Stroop Test: the effects of reaction mode, order and practice. *The Japanese Journal of Educational Psychology*.

[28] Takeuchi H., Taki Y., Sassa Y. (2012). Regional gray and white matter volume associated with stroop interference: evidence from voxel-based morphometry. *NeuroImage*.

[29] Society For Creative Minds (1969). *Manual of S-A Creattvity Test*.

[30] Takeuchi H., Taki Y., Sassa Y. (2010). Regional gray matter volume of dopaminergic system associate with creativity: evidence from voxel-based morphometry. *NeuroImage*.

[31] Takeuchi H., Taki Y., Sassa Y. (2010). White matter structures associated with creativity: evidence from diffusion tensor imaging. *NeuroImage*.

[32] Klingberg T., Fernell E., Olesen P. J. (2005). Computerized training of working memory in children with ADHD-a randomized, controlled trial. *Journal of the American Academy of Child & Adolescent Psychiatry*.

[33] Klingberg T., Forssberg H., Westerberg H. (2002). Training of working memory in children with ADHD. *Journal of Clinical and Experimental Neuropsychology*.

[34] Necka E. (1999). Creativity and attention. *Polish Psychological Bulletin*.

[35] Mahncke H. W., Connor B. B., Appelman J. (2006). Memory enhancement in healthy older adults using a brain plasticity-based training program: a randomized, controlled study. *Proceedings of the National Academy of Sciences of the United States of America*.

[36] Jaeggi S. M., Buschkuehl M., Jonides J., Shah P. (2011). Short- and long-term benefits of cognitive training. *Proceedings of the National Academy of Sciences of the United States of America*.

[37] Tang Y.-Y., Ma Y., Wang J. (2007). Short-term meditation training improves attention and self-regulation. *Proceedings of the National Academy of Sciences of the United States of America*.

[38] Rueda M. R., Rothbart M. K., McCandliss B. D., Saccomanno L., Posner M. I. (2005). Training, maturation, and genetic influences on the development of executive attention. *Proceedings of the National Academy of Sciences of the United States of America*.

[39] Erickson K. I., Voss M. W., Prakash R. S. (2011). Exercise training increases size of hippocampus and improves memory. *Proceedings of the National Academy of Sciences of the United States of America*.

[40] Neville H. J., Stevens C., Pakulak E. (2013). Family-based training program improves brain function, cognition, and behavior in lower socioeconomic status preschoolers. *Proceedings of the National Academy of Sciences of the United States of America*.

[41] Anderson S., White-Schwoch T., Parbery-Clark A., Kraus N. (2013). Reversal of age-related neural timing delays with training. *Proceedings of the National Academy of Sciences of the United States of America*.

[42] Benjamini Y., Krieger A. M., Yekutieli D. (2006). Adaptive linear step-up procedures that control the false discovery rate. *Biometrika*.

[43] Jaeggi S. M., Buschkuehl M., Jonides J., Perrig W. J. (2008). Improving fluid intelligence with training on working memory. *Proceedings of the National Academy of Sciences of the United States of America*.

[44] Petersen E. T., Lim T., Golay X. (2006). Model-free arterial spin labeling quantification approach for perfusion MRI. *Magnetic Resonance in Medicine*.

[45] Petersen E. T., Mouridsen K., Golay X. (2010). The QUASAR reproducibility study, part II: results from a multi-center arterial spin labeling test-retest study. *NeuroImage*.

[46] Petersen E. T., Zimine I., Ho Y.-C. L., Golay X. (2006). Non-invasive measurement of perfusion: a critical review of arterial spin labelling techniques. *British Journal of Radiology*.

[47] Taki Y., Hashizume H., Sassa Y. (2011). Correlation between gray matter density-adjusted brain perfusion and age using brain MR images of 202 healthy children. *Human Brain Mapping*.

[48] Driemeyer J., Boyke J., Gaser C., Büchel C., May A. (2008). Changes in gray matter induced by learning—revisited. *PLoS ONE*.

[49] Gaser C. VBM Toolbox for SPM2, VBM Toolbox for SPM5. http://www.neuro.uni-jena.de/.

[50] Reuter M., Fischl B. (2011). Avoiding asymmetry-induced bias in longitudinal image processing. *NeuroImage*.

[51] Takeuchi H., Taki Y., Sassa Y. (2011). Regional gray matter density associated with emotional intelligence: evidence from voxel-based morphometry. *Human Brain Mapping*.

[52] Ashburner J., Friston K. J. (2000). Voxel-based morphometry—the methods. *NeuroImage*.

[53] Takeuchi H., Taki Y., Nouchi R. (2012). A voxel-based morphometry study of gray and white matter correlates of a need for uniqueness. *NeuroImage*.

[54] Hayasaka S., Phan K. L., Liberzon I., Worsley K. J., Nichols T. E. (2004). Nonstationary cluster-size inference with random field and permutation methods. *NeuroImage*.

[55] Silver M., Montana G., Nichols T. E. (2012). False positives in neuroimaging genetics using voxel-based morphometry data. *NeuroImage*.

[56] Takeuchi H., Taki Y., Hashizume H. (2011). Cerebral blood flow during rest associates with general intelligence and creativity. *PLoS ONE*.

[57] Takeuchi H., Sekiguchi A., Taki Y. (2010). Training of working memory impacts structural connectivity. *Journal of Neuroscience*.

[58] Casanova R., Srikanth R., Baer A. (2007). Biological parametric mapping: a statistical toolbox for multimodality brain image analysis. *NeuroImage*.

[59] Friston K. J., Holmes A., Poline J.-B., Price C. J., Frith C. D. (1996). Detecting activations in PET and fMRI: levels of inference and power. *NeuroImage*.

[60] Dreher J.-C., Koechlin E., Tierney M., Grafman J. (2008). Damage to the fronto-polar cortex is associated with impaired multitasking. *PLoS ONE*.

[61] Quallo M. M., Price C. J., Ueno K. (2009). Gray and white matter changes associated with tool-use learning in macaque monkeys. *Proceedings of the National Academy of Sciences of the United States of America*.

[62] May A., Hajak G., Gänssbauer S. (2007). Structural brain alterations following 5 days of intervention: dynamic aspects of neuroplasticity. *Cerebral Cortex*.

[63] Takeuchi H., Taki Y., Nouchi R. (2013). Effects of working memory training on functional connectivity and cerebral blood flow during rest. *Cortex*.

[64] Huttenlocher P. R., Dabholkar A. S. (1997). Regional differences in synaptogenesis in human cerebral cortex. *The Journal of Comparative Neurology*.

[65] Trachtenberg J. T., Chen B. E., Knott G. W. (2002). Long-term in vivo imaging of experience-dependent synaptic plasticity in adult cortex. *Nature*.

[66] Xu T., Yu X., Perlik A. J. (2009). Rapid formation and selective stabilization of synapses for enduring motor memories. *Nature*.

[67] Butterworth B., Walsh V. (2011). Neural basis of mathematical cognition. *Current Biology*.

[68] Sirevaag A. M., Greenough W. T. (1987). Differential rearing effects on rat visual cortex synapses. III. Neuronal and glial nuclei, boutons, dendrites, and capillaries. *Brain Research*.

[69] Borowsky I. W., Collins R. C. (1989). Metabolic anatomy of brain: a comparison of regional capillary density, glucose metabolism, and enzyme activities. *Journal of Comparative Neurology*.

[70] Johansen-Berg H., Baptista C. S., Thomas A. G. (2012). Human structural plasticity at record speed. *Neuron*.

[71] Hogarty G. E., Flesher S., Ulrich R. (2004). Cognitive enhancement therapy for schizophrenia: effects of a 2-year randomized trial on cognition and behavior. *Archives of General Psychiatry*.

[72] Takeuchi H., Taki Y., Kawashima R. (2010). Effects of working memory training on cognitive functions and neural systems. *Reviews in the Neurosciences*.

[73] Green C. S., Bavelier D. (2003). Action video game modifies visual selective attention. *Nature*.

[74] Hayasaka S., Nichols T. E. (2003). Validating cluster size inference: random field and permutation methods. *NeuroImage*.

[75] Christoff K., Gabrieli J. D. E. (2000). The frontopolar cortex and human cognition: evidence for a rostrocaudal hierarchical organization within the human prefrontal cortex. *Psychobiology*.

