# Supplementary material for: Effects of Fast Simple Numerical Calculation Training on Neural Systems
Source: Neural Plast. 2016 Jan 6;2016:5940634. doi: 10.1155/2016/5940634 (PMC4736604; doi:10.1155/2016/5940634)

Supplemental online material.

Supplemental Table 1. Pre- and post-test scores for the psychological measures of the active control group from a previous study [1](Mean ± SEM)

	Active control		Planned Contrast in ANCOVA[c]	*P* value[c] (uncorrected, corrected[e])
	pre	post		
Arithmetic				
Simple arithmetic	33.0 ± 1.4	35.4 ± 0.9	FSNC training >2 control[d]	$7.31*10^{-4}$, 0.005
Complex arithmetic	7.06 ± 0.54	7.38 ± 0.67	FSNC training >2 control	0.034, 0.059
Non-verbal reasoning				
RAPM[a] (score)	29.1 ± 0.9	32.0 ± 0.8	FSNC training >2 control	0.270, 0.210
CCFT[b] (score)	No data	No data	FSNC training >1 control	0.754, 0.440
Working memory (WM)				
Digit span (score)	No data	No data	FSNC training >1 control	0.702, 0.440
Visuospatial WM	No data	No data	FSNC training >1 control	0.241, 0.210
Intelligence test with				
Tanaka B type	No data	No data	FSNC training >1 control	0.301, 0.211
Simple processing speed				
Word-Color task (items)	73.1 ± 1.7	79.3 ± 1.5	FSNC training >2 control	0.235, 0.210
Color-Word task (items)	52.9 ± 1.8	55.9 ± 1.6	FSNC training >2 control	0.018, 0.042
Executive function				
reverse Stroop task	62.0 ± 2.2	66.6 ± 1.6	FSNC training >2 control	0.009, 0.032
Stroop task (items)	49.0 ± 1.7	51.2 ± 1.5	FSNC training >2 control	0.061, 0.085
Creativity				
S-A creativity test (total	26.9 ± 1.7	27.3 ± 1.5	two-tailed (FSNC training vs. 2 controls)	0.109, 0.127

grade)

Data are reported as the mean ± SEM

a. Raven's Advanced Progressive Matrices

b. Cattell's Culture Fair Test

c. One-way ANCOVAs with test–retest differences were performed with psychological measures as dependent variables and pretest scores on the psychological measures as covariates. When there were data from three control groups, the comparisons were performed between the FSNC training group and the three control groups. When there were data from only one control group, the comparisons were performed between the FSNC training group and the control group.

d. The no-intervention group in the experiment of the present study and the active control group from the previous study.

e. P values of results that were corrected for multiple comparisons using FDR.

Supplemental Figure legend

Supplemental Fig. 1. Schema of tasks used in this study. There were two control tasks (a word-color task and a color-word task), a reverse Stroop task, and a Stroop task. This figure is reproduced from our previous publication [2].

Supplemental Fig. 2. Areas of analyses of regional cerebral blood flow during rest (resting rCBF). Because of the area limitations of the scanning method, only parts of brain were scanned and analyzed. Black regions are areas analyzed for resting rCBF, as displayed on the glass brain.

References

1. Takeuchi H, Taki Y, Sassa Y, Hashizume H, Sekiguchi A, et al. (2011) Working memory training using mental calculation impacts regional gray matter of the frontal and parietal regions. PLoS ONE 6: e23175.

2. Takeuchi H, Taki Y, Sassa Y, Hashizume H, Sekiguchi A, et al. (2012) Regional gray and white matter volume associated with Stroop interference: Evidence from voxel-based morphometry. Neuroimage 59: 2899-2907.

Supplemental Fig. 1.

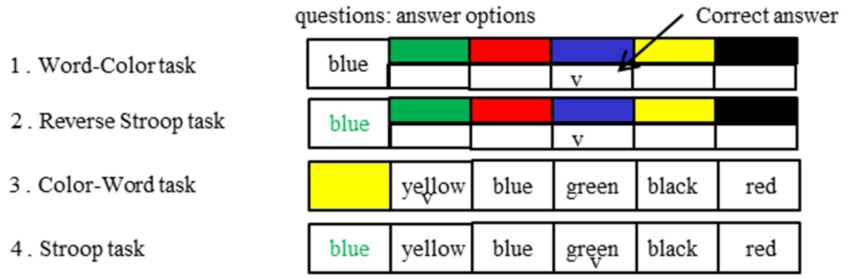

Supplemental Fig. 2.

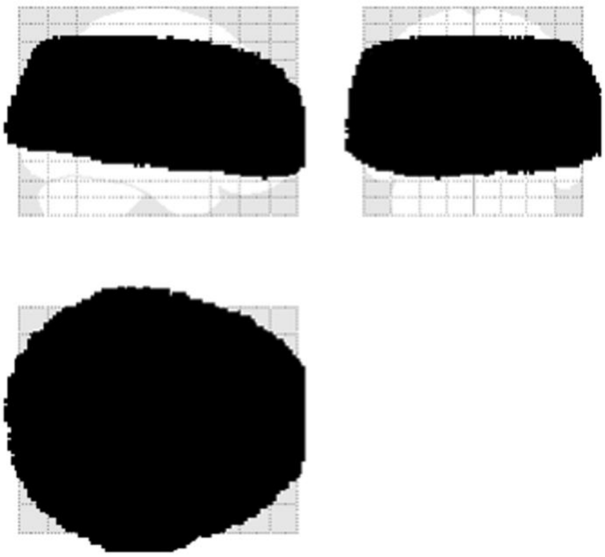

Supplement: Supplementary file 1 — Supplementary materials contain the table which described the data of the active control group in our previous study (Supplemental Table 1), the figure which showed the schema of the stroop tasks that we used (Supplemental Figure 1), and the figure which showed the areas of regional cerebral blood flow during rest (Supplemental figure 2). [file 5940634.f1.pdf]
